# Clinical Features, Treatment, and Surveillance of Hyperparathyroidism-Jaw Tumor Syndrome: An Up-to-Date and Review of the Literature

**DOI:** 10.1155/2019/1761030

**Published:** 2019-12-18

**Authors:** Francesca Torresan, Maurizio Iacobone

**Affiliations:** Endocrine Surgery Unit, Department of Surgery, Oncology and Gastroenterology, University of Padova, Via Giustiniani 2, 35128 Padova, Italy

## Abstract

Hyperparathyroidism-jaw tumor (HPT-JT) syndrome is an autosomal dominant disorder characterized by parathyroid tumors in association with fibro-osseous jaw tumors and uterine and renal lesions. HPT-JT syndrome is caused by germline mutations of the cell division cycle 73 (*CDC73*) gene that encodes the parafibromin, a 531-amino acid protein with antiproliferative activity. Primary hyperparathyroidism is the main finding of HPT-JT syndrome, usually caused by a single-gland parathyroid involvement (80% of cases), at variance with other variants of hereditary hyperparathyroidism, in which a multiglandular involvement is more frequent. Moreover, parathyroid carcinoma may occur in approximately 20% of cases. Surgery is the treatment of choice for primary hyperparathyroidism, but the extent of surgery remains controversial, varying between bilateral neck and focused exploration, with subtotal or limited parathyroidectomy. Recently, more limited approaches and parathyroid excisions have been suggested in order to decrease the risk of permanent hypoparathyroidism, the main surgical morbidity following more extensive surgical approaches. Ossifying fibromas of the mandible or maxilla may present only in a minority of cases and, even if benign, they should be surgically treated to avoid tumor growth and subsequent functional limitations. Benign and malignant uterine involvement (including leiomyomas, endometrial hyperplasia, adenomyosis, multiple adenomyomatous polyps, and adenosarcomas) is the second most common clinical feature of the syndrome, affecting more than 50% of *CDC73*-carrier women. Genetic testing should be performed in all family members of affected individuals, in young patients undergoing surgery for primary hyperparathyroidism, or in presence of other associated tumors, allowing early diagnosis and prompt treatment with more tailored surgery. Moreover, *CDC73* mutation carriers should be also periodically screened for primary hyperparathyroidism and the other associated tumors. The present review was aimed to summarize the main clinical features of HPT-JT syndrome, focusing on genetic screening and surgical treatment, and to revise the available literature.

## 1. Introduction

Hyperparathyroidism-jaw tumor syndrome (HPT-JT) (OMIM#145001) is a rare autosomal dominant disorder with incomplete penetrance characterized by the development of parathyroid tumors, ossifying fibromas of the mandible and maxilla, cystic and neoplastic renal abnormalities, and hyperplastic and neoplastic uterine involvement [1, 2].

HPT-JT syndrome is caused by germline mutations of the *CDC73* gene that encodes the parafibromin, a ubiquitously expressed, predominantly nuclear protein with antiproliferative properties [3–6].

Despite the nomenclature of the syndrome, jaw tumors may be found only in approximately one third of cases, while the most common, and sometimes the only feature of HPT-JT, is primary hyperparathyroidism (pHPT). At variance with other forms of hereditary pHPT in which parathyroid tumors are generally benign, HPT-JT is associated with a higher prevalence of atypical adenomas and carcinomas [7, 8].

Genetic testing is required to confirm the hereditary nature of pHPT in HPT-JT, and it is crucial for the optimal clinical and surgical management of the affected individuals; moreover, a molecular genetic testing should be obtained in at risk relatives in order to identify the gene carriers as early as possible to start surveillance and early treatment [9].

The aim of this review is to summarize the current knowledge on HPT-JT syndrome including clinical features, genetic, and treatments and to revise the available literature.

## 2. Etiology and Diagnosis

HPT-JT is linked to germline-inactivating mutations in the tumor suppressor gene *CDC73* (formerly *HRPT2*), which is comprised of 17 exons on chromosome 1q31.2 and encodes for the predominantly nuclear, 531-amino acid protein, parafibromin [10]. Parafibromin is ubiquitously expressed in a variety of human tissues, including kidney, liver, stomach, renal cortex tubules, and the pars intermedia of the hypophysis [11].

Parafibromin is associated with other proteins in the polymerase-associated factor (PAF1) and induces the downregulation of cyclin D1 expression and direct interaction with *ß*-catenin, resulting in the activation of transcription of target genes. Studies of the PAF1 complex in yeast and *Drosophila*, as well as in mammalian cells, have revealed that parafibromin, as part of the PAF1 complex, induces histone modification, transcription elongation, and chromatin remodeling [12–14].

About 75% of HPT-JT patients have germline *CDC73* mutations within the coding region, and the majority (>80%) are frameshift or nonsense mutations that determine the functional loss of parafibromin by causing a premature truncation of this protein or a rapid loosing of the translated protein via nonsense-mediated mRNA decay. Therefore, the expression of parafibromin is completely lost in HPT-JT-associated tumor tissues. Immunohistochemical staining of parafibromin in parathyroid tumors and other HPT-JT-associated tumors is an indirect method to recognize HPT-JT syndrome patients. The remaining 25% of HPT-JT patients may have abnormalities in *CDC73* promoter regions, whole exon or gene deletions, mutations in unidentified genes, or epigenetic modifications [15].

As in all tumor suppressor genes, the first mutation is usually inherited by one of the parents or, in very rare cases, developed *de novo* at embryo level; the second allele-inactivating mechanism is a novel acquired somatic mutation or a loss of heterozygosity in HPT-JT tumor-related tissues, consistent with Knudson's two-hit hypothesis [7–16].

Even in presence of a *CDC73* mutation, the penetrance of pHPT is incomplete and no genotype-phenotype correlations have been fully established to date. However, it has been suggested that missense mutations are more likely to be associated with the disease without typical associated features (familial isolated pHPT), whereas mutations causing gross parafibromin disruption are more likely associated with the classical HPT-JT phenotype [9].

The diagnosis of HPT-JT must be confirmed by genetic testing. The screening for *CDC73* germline mutations is indicated in presence of familial pHPT, in case of pHPT with young age onset (<40 years), multiglandular involvement, cystic, atypical, or malignant parathyroid involvements, or in presence of coexistence ossifying jaw fibroma, renal, or uterine tumors [9, 17]. As in the original description of HPT-JT syndrome, where it was characterized as cystic parathyroid adenomatosis, the adenomas may be cystic, either with micro- or macrocysts, and similar cystic changes can also be present in the normal parathyroid glands in these patients [18].

Following the initial diagnosis, the associated jaw tumors and renal and uterine lesions should be systematically searched [3–6].

Genetic screening for identifying the gene carrier and/or affected family members should be performed in all family members to start a specific HPT-JT screening program [19]. In *CDC73* mutation carrier families, the screening should be performed also in children before the age of 10 since malignant pHPT has been sometimes described at very early age.

## 3. Clinical Features

### 3.1. pHPT

pHPT is the main finding of HPT-JT syndrome and is found in almost 100% of mutation carriers typically in late adolescence or early adulthood. The earliest reported age of hypercalcemia is seven years [20]. The median age of diagnosis of pHPT reported was 27 years (range 12–58) [21], and the mean age ranged between 32 years and 36 years [4, 22]. In a report of a three large kindred, *CDC73*-related pHPT occurred in 87.5% of cases among patients older than 20 years [9], while penetrance of pHPT in a Dutch population was shown to increase with age (8%, 53%, and 75% at ages 25, 50, and 70, respectively) [22]. To date, 154 HPT-JT kindreds have been reported in the literature (Table 1), including 365 patients affected by pHPT. At variance with other forms of hereditary pHPT, in HPT-JT, a single-gland parathyroid involvement has been reported more frequently (86.1%). Multiglandular involvement occurs rarely at initial surgery (13.9% of cases); recurrences of pHPT may occur metachronously in 20% of cases. At pathology, a single benign parathyroid adenoma is usually found; however, parathyroid carcinoma may be found in 23% of cases [2–82].

pHPT is usually mild and/or asymptomatic, but, in the case of parathyroid carcinoma, severe hypercalcemic crises may occur [83]. Hence, in presence of abnormal high serum calcium concentration (>12 mg/dL) and iPTH levels (>3 times the upper limit of normal) and parathyroid lesions larger than 3 cm, parathyroid carcinomas should be suspected, even if nonfunctioning parathyroid malignancy may very rarely occur in *CDC73*-related disorder [35, 82]. Moreover, parathyroid carcinoma can present as palpable neck mass associated with hoarseness, difficulty speaking or swallowing, muscle weakness, nausea/vomiting, altered mental status, bone pain, and/or pathologic bone fractures [7, 17, 22, 23, 38].

Given the rarity of the disease and the heterogeneity of the phenotype, the optimal surgical approach to *CDC73*-related pHPT has not yet been established and remains controversial, varying between bilateral or targeted neck exploration and extensive or limited parathyroidectomy [9].

In the past, prophylactic subtotal or total parathyroidectomy has been suggested to reduce the risk of recurrences and parathyroid carcinoma in HPT-JT syndrome and therefore to obtain a definitive cure; however, total parathyroidectomy is clearly not always successful and leads to an increased morbidity due to the permanent postsurgical hypoparathyroidism that is difficult to treat especially in young patients. For these reasons, subtotal parathyroidectomy or total parathyroidectomy with autotransplantation has been suggested in the same setting of other variants of hereditary pHPT, even if autotransplantation has been advocated to theoretically cause tumor dissemination in case of malignant involvement [82]. Moreover, because of the reported high prevalence of uniglandular involvement at onset, targeted approaches and selective parathyroid excisions have recently been proposed in order to achieve, whenever possible, the longest possible normocalcemia without permanent hypoparathyroidism, minimizing surgical morbidity and facilitating eventual future surgery for recurrent disease [4].

In 2008, Sarquis et al. have observed, in a series of 11 *CDC73* germline mutated patients from 3 kindreds, a synchronous multiglandular involvement at initial operation in 54.5% of cases, parathyroid malignancy in 9%, and an overall persistence/recurrence rate of 80%; thus, a bilateral exploration with subtotal parathyroidectomy was suggested as the initial approach [41].

In 2014, Mehta et al. suggested a bilateral neck exploration with selective removal only of abnormal gland/s in HPT-JT syndrome patients, given the high frequency of benign single-gland involvement (69%) and relatively low rate of reoccurences (20%) found in their multicentric cohort of 16 individuals from seven families [62].

More recently, Iacobone et al. reported, in a cohort of 20 HPT-JT syndrome patients from five large families, a 95% rate of single-gland involvement. Therefore, in case of preoperative imaging techniques localizing concordantly, a single-gland involvement and in absence of suspicion of parathyroid malignancy, a focused approach with selective parathyroidectomy has been proposed. In case of absent or discordant preoperative localization, a subtotal parathyroidectomy was suggested because of the increased risk of multiglandular involvement and recurrent pHPT [82]. Regardless of the surgical approach, the risk of recurrences is estimated, according to the review of the literature (Table 1), to be about 25%, thus requiring a regular lifelong pHPT biochemical and instrumental monitoring.

If parathyroid carcinoma is clinically suspected (large tumor at imaging, palpable neck mass, and biochemical and clinical presentation of severe pHPT), an en bloc resection of the mass with the ipsilateral thyroid lobe, possibly also including the ipsilateral normal parathyroid and the surrounding lymph fatty tissue, should be performed, in order to avoid tumor seeding and achieve a “complete unilateral parathyroidectomy,” and finally avoiding the risk of reoperation in a scarred area [4, 82–84].

Cinacalcet hydrochloride, a calcimimetic that binds to the calcium-sensing receptor, has been approved for the long-term control of hypercalcemia secondary to pHPT in individuals who are unable to undergo parathyroidectomy and for the treatment of parathyroid carcinoma-related hypercalcemia, in case of unresectable or metastatic disease.

For severe or symptomatic hypercalcemia, individuals can be treated with an infusion of zoledronic acid or denosumab for acute management.

### 3.2. Jaw Tumors

Jaw tumors in HPT-JT are fibro-osseous lesions that typically involve the maxilla or mandible that occur in about 30% of cases (Table 1), often prior to the third decade of life.

The majority of the reported jaw tumors in HPT-JT syndrome are ossifying fibromas, benign and generally slow-growing tumors arising from the periodontal ligament in molar or premolar areas [85]. Even if specific features of ossifying fibroma in HPT-JT syndrome are not well defined, most often they appear to be radiographically radiolucent compared to the mixed radiolucent/radiopaque lesions in the sporadic variants [32]. They may present as an enlarging visible or palpable mass, or in some cases, they are only detected on dental X-ray imaging.

Although benign, ossifying fibroma can disrupt normal dentition and impair breathing, causing functional and cosmetic symptoms. Moreover, ossifying fibroma in HPT-JT syndrome may be bilateral/multifocal and may recur. For these reasons, complete surgical removal is the recommended treatment based on the size, location, and symptoms of the lesion. Individuals with a history of jaw tumors should be followed closely because of the possibility of recurrence [86].

### 3.3. Renal Involvement

The kidney is involved in a limited subset of patients with HPT-JT (approximately 15% of cases). Cystic kidney disease is the most common renal manifestation of this syndrome.

In addition to renal cysts, some patients often develop hamartomas and rare renal tumors, such as adult Wilms's tumors and mixed epithelial-stromal tumors (MEST). Wilms's tumors in HPT-JT have been identified in the fifth decade of life, are usually bilateral, poorly circumscribed, of smaller sizes compared to the classical childhood form, and they usually do not metastasize. Moreover, they had also distinctive histological features from the childhood form, such as a low number of mitoses, lack of necrosis and hemorrhages, large mesenchymal components, and the presence of cysts [87].

The association between MEST, a predominantly benign tumor characterized by both epithelial and spindle cell stromal components, and HPT-JT syndrome is poorly reported in the literature.

Since HPT-JT patients may be at risk for multiple, bilateral renal tumors potentially requiring multiple renal surgeries over their lifetime, nephron-sparing surgery rather than radical surgery is advocated, in order to preserve renal function [88]. Moreover, given the malignant potential, sarcomatoid differentiation, and metastatic spread, surgery represents the treatment of choice [89].

Papillary renal cell carcinoma have also been described, albeit rarely, in HPT-JT [4, 90].

### 3.4. Uterine Involvement

Uterine tumors have been described in association with HPT-JT and are the most common clinical feature after pHPT, affecting more than 50% of HPT-JT female patients in some cohorts [9].

Most women present with menorrhagia that may require hysterectomy at an early age (mean 35 years). Affected women often have a history of miscarriage and a significantly impaired ability to bear children when compared with their unaffected female relatives [33]. Histological analysis of the uterine specimens revealed both benign and malignant tumors, such as adenomyosis, adenofibromas, leiomyomas, endometrial hyperplasia, adenosarcomas, or tumors arising from the Müllerian duct system.

No treatment guidelines for uterine manifestations associated with HPT-JT syndrome have been proposed to date. Individuals with evidence of a uterine tumor should be managed by a gynecologist on a case-by-case basis.

### 3.5. Other Features

Thyroid carcinoma, thyrotoxicosis, colon carcinoma, cholangiocarcinoma, chronic lymphatic leukemia, pancreatic adenocarcinoma, and pituitary cyst have also been described, but the association between these less common tumors and HPT-JT syndrome remains unclear [4, 42, 90].

### 3.6. Surveillance

Even if there are no well-established surveillance guidelines, according to the literature we suggest that *CDC73* mutation carriers should undergo the following screening:

- Biannual evaluation of serum calcium and PTH for pHPT screening, possibly starting at the age of five, and periodic parathyroid ultrasound examination
- Panoramic X-ray dental imaging at least every five years
- Monitor for kidney lesions by periodic renal ultrasound examination, magnetic resonance imaging, or computed tomography scan at least every 5 years, starting at the age of diagnosis
- Starting at the reproductive age, women with a *CDC73*-related disorder should undergo regular gynecologic care, including pelvic ultrasound examination with eventually further imaging studies if clinically indicated.

## Figures and Tables

**Table 1:** Review of the literature focusing on HPT-JT syndrome.

Author (year)	Kindred (*n*=)	pHPT (*n*=)	Single-gland pHPT (*n*=)	Synchronous multiglandular pHPT (*n*=)	Recurrences (*n*=)	Parathyroid carcinoma (*n*=)	Jaw tumor (*n*=)	Renal lesions (*n*)	Uterine lesions (*n*)
Carpten et al. (2002) [7]	14	66	NA	NA	NA	11	30	18	NA
Shattuck et al. (2003) [23]	3	3	NA	NA	NA	3	NA	NA	NA
Howell et al. (2003) [24]	3	7	NA	NA	0	3	0	0	NA
Simonds et al. (2004) [25]	1	4	4	0	0	1	0	0	NA
Cetani et al. (2004) [17]	2	4	3	1	NA	0	0	0	NA
Villablanca et al. (2004) [26]	2	9	7	2	3	0	0	0	NA
Cavaco et al. (2004) [27]	6	9	5	1	0	0	2	2	NA
Howell et al. (2004) [28]	1	2	2	0	NA	0	1	NA	NA
Bradley et al. (2005) [2]	2	9	NA	NA	NA	2	11	0	6
Moon et al. (2005) [29]	1	2	2	0	NA	2	1	NA	NA
Gimm et al. (2006) [30]	1	3	1	1	1	1	NA	NA	NA
Mizusawa et al. (2006) [31]	3	7	6	0	1	1	1	0	0
Aldred et al. (2006) [32]	1	3	3	0	0	0	2	NA	NA
Bradley et al. (2006) [33]	5	5	4	1	NA	0	2	0	1
Juhlin et al. (2006) [34]	1	1	1	NA	NA	0	NA	NA	NA
Guarnieri et al. (2006) [35]	1	4	4	0	1	1	NA	0	2
Kelly et al. (2006) [8]	1	3	2	1	2	2	NA	NA	NA
Yamashita et al. (2007) [36]	1	1	1	0	0	0	1	NA	NA
Cetani et al. (2007) [37]	1	1	1	0	1	0	0	0	NA
Cetani et al. (2007) [38]	2	3	NA	NA	NA	3	NA	NA	NA
Raue et al. (2007) [39]	1	2	1	1	NA	1	1	NA	NA
Cetani et al. (2008) [40]	1	1	1	0	NA	1	0	NA	NA
Sarquis et al. (2008) [41]	3	11	5	6	6	1	1	4	5
Guarnieri et al. (2008) [42]	3	3	3	0	1	3	0	3	3
Howell et al. (2009) [43]	1	1	1	0	0	NA	NA	NA	NA
Silveira et al. (2008) [44]	1	9	3	6	6	1	0	4	5
Schmidt et al. (2009) [45]	1	1	1	0	0	0	1	NA	NA
Rekik et al. (2010) [46]	1	1	1	0	0	0	1	0	1
Panicker et al. (2010) [47]	1	5	NA	NA	NA	0	1	0	1
Veiguela et al. (2010) [48]	1	7	NA	NA	NA	1	3	0	2
Cavaco et al. (2011) [49]	2	2	2	0	1	2	0	0	0
Pichardo-Lowden et al. (2011) [20]	1	1	1	0	1	0	0	1	NA
Frank-Raue et al. (2011) [50]	7	11	9	1	1	3	8	0	2
Cascón et al. (2011) [10]	1	3	NA	NA	NA	0	3	NA	NA
Siu et al. (2011) [51]	2	2	2	0	0	1	0	0	NA
Domingues et al. (2012) [52]	1	1	1	0	0	0	0	0	NA
Guerrouani et al. (2013) [53]	1	1	1	0	NA	0	1	NA	NA
Bricaire et al. (2013) [21]	NA	19	NA	NA	NA	5	4	4	6
Kutcher et al. (2013) [54]	1	1	0	1	0	1	1	1	NA
Ghemigian et al. (2013) [55]	1	3	3	0	0	0	0	NA	NA
Abdulla et al. (2013) [56]	1	1	0	1	1	0	1	NA	NA
Pazienza et al. (2013) [57]	3	7	7	0	0	0	0	1	1
Kong et al. (2014) [58]	1	2	0	2	1	0	1	NA	2
Chiofalo et al. (2014) [59]	1	2	2	0	0	1	1	0	NA
Korpi-Hyövälti et al. (2014) [60]	1	7	NA	NA	NA	2	NA	2	NA
Sriphrapradang et al. (2014) [61]	1	1	1	0	NA	1	1	0	NA
Mehta^a^ et al. (2014) [62]	7	16	11	5	4	6	2	3	2
Parfitt et al. (2015) [63]	1	1	1	0	0	1	1	0	0
Shibata et al. (2015) [64]	1	1	1	0	1	0	0	0	0
Khadilkar et al. (2015) [65]	4	6	6	0	2	1	2	3	2
Marchiori et al. (2015) [66]	1	1	1	0	0	1	1	0	0
Bellido et al. (2016) [67]	1	1	1	0	0	0	1	NA	NA
Ennazk et al. (2016) [68]	1	1	1	0	0	0	1	NA	NA
Mathews et al. (2016) [69]	1	1	1	0	0	0	1	NA	NA
Mele et al. (2016) [70]	1	1	1	0	1	1	1	NA	0
Piciu et al. (2016) [71]	1	1	1	0	0	0	1	0	1
Guarnieri et al. (2017) [72]	1	3	3	0	0	1	0	0	1
Mamedova et al. (2017) [73]	6	6	6	0	1	4	0	0	0
van der Tuin et al. (2017) [22]	12	32	32	0	NA	5	6	10	1
Dhas et al. (2017) [74]	1	1	1	0	0	0	1	0	1
Rubinstein et al. (2017) [75]	1	1	1	0	0	0	0	0	1
Koikawa et al. (2018) [76]	1	1	1	0	0	0	1	0	1
Bachmeier et al. (2018) [77]	1	1	1	0	0	0	0	0	0
Kapur et al. (2018) [78]	1	1	1	0	NA	1	0	0	0
Ciuffi et al. (2019) [79]	1	1	1	0	NA	1	1	NA	0
Russo et al. (2019) [80]	1	1	1	0	1	1	1	NA	NA
Gill et al. (2019) [81]	13	16	15	1	3	4	1	NA	NA
Iacobone et al. (2019) [82]	5	20	19	1	6	3	2	1	14
Total *n*= (%)	154	365	198 (86.1%)	32 (13.9%)	46 (20%)	84 (23%)	104 (28.4%)	57 (15.6%)	60 (45.1%)

^a^Some cases have been previously included in Carpten et al. [7]. NA = not available; pHPT =  primary hyperparathyroidism.
